# Nuclear myocardial perfusion imaging in stable angina pectoris: Sometimes being wrong is all right

**DOI:** 10.1007/s12471-018-1105-5

**Published:** 2018-03-09

**Authors:** A. Dedic, R. L. Braam

**Affiliations:** 1 Department of Cardiology, Erasmus MC, Rotterdam, The Netherlands; 2 Department of Cardiology, Gelre Hospitals, Apeldoorn, The Netherlands

Every day physicians balance clinical information with medical test results when confronted with symptomatic patients. Sometimes they find themselves in an apparently contradicting situation in which a patient has persistent complaints while his or her medical tests are normal. In this issue of the Netherlands Heart Journal, Yokota et al. addressed this matter in the setting of stable angina pectoris [1]. The authors performed a retrospective analysis of all patients who had undergone nuclear myocardial perfusion imaging in their centre and selected those with a normal scan but with persistent or worsening complaints that compelled the treating physician to order an invasive angiogram. Out of more than 11,000 patients, 229 fulfilled the study criteria.

The authors reported that in this highly selected group of patients a fairly high percentage (34%) had significant coronary artery disease despite a normal perfusion scan, which was defined as >50% stenosis in the left main coronary artery or >70% stenosis for other segments. In the majority of cases, it concerned single-vessel disease (60%), while only a minority (17%) had left main coronary artery disease or three-vessel disease. Coronary revascularisation was performed in 90% and most of them were free of symptoms after 7 years of follow-up. The authors found that older age, male sex, typical angina and previous PCI are independent predictors for the presence of severe stenosis on invasive angiography following a normal myocardial perfusion scan. As the study was conducted in a ‘pre-FFR era’ there was a low rate of invasive functional testing, which in part might explain the discordancy.

This study provides us with new insights on the diagnostic value of nuclear myocardial perfusion imaging and refutes the common belief of balanced ischaemia (three-vessel or left main coronary artery disease) as the reason for false-negative perfusion scans. Assuming that the scans were performed according to the modern technological standards and known pitfalls as the use of xanthine derivate were avoided, no other reason than a shortcoming of the test itself can be put forward; no test is perfect.

Most importantly, this study shows the importance of assessing the pre-test probability of disease in clinical practice. Looking at the patient characteristics of this highly selected group, the authors examined a high-risk population, which is also reflected in the 14% mortality after 7 years of follow-up.

**Fig. 1:**
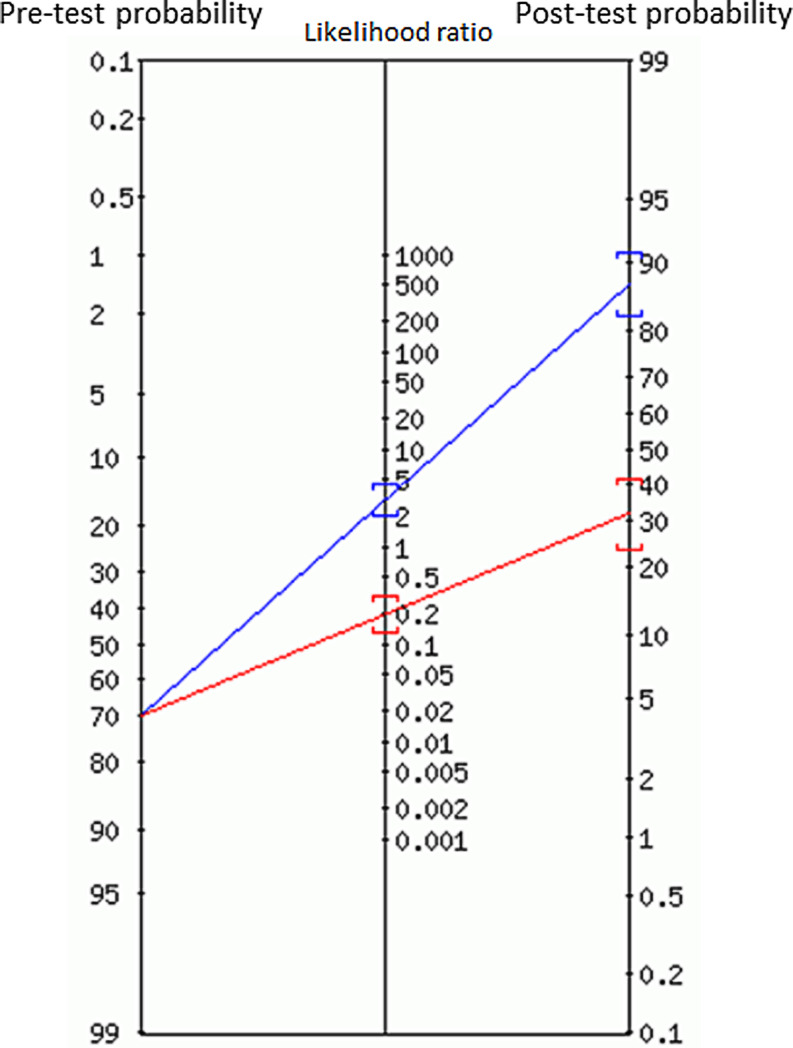
A patient with a pre-test probability of disease of 70% who undergoes a test with a sensitivity of 86% and a specificity of 72% will have a post-test probability of disease of 32% in case of a negative test and 88% with a positive test

When a diagnostic test with a sensitivity between 85–90% and a specificity around 70% is employed in this population, the reported ‘false-negative’ rate of 34% is not surprising (Fig. 1; [2]). Patients with angina pectoris and a high pre-test probability should be considered to have significant coronary artery disease on forehand and do not actually need further testing for the diagnosis itself. Performing a non-invasive test in these patients is nevertheless sensible as it provides valuable prognostic information. Patients with extensive ischaemia will benefit from a proactive coronary revascularisation strategy while those with a normal test can be treated with medical therapy [3]. There are sufficient data that show that a normal nuclear myocardial perfusion scan is associated with a favourable prognosis [4]. When complaints persist or worsen despite appropriate medical therapy, further invasive testing should be considered, especially in those with advanced age, male gender or previous PCI as these are associated with high risk, and a false-negative scan is very much possible.
